# A gene expression-based study on immune cell subtypes and glioma prognosis

**DOI:** 10.1186/s12885-019-6324-7

**Published:** 2019-11-15

**Authors:** Qiu-Yue Zhong, Er-Xi Fan, Guang-Yong Feng, Qi-Ying Chen, Xiao-Xia Gou, Guo-Jun Yue, Gui-hai Zhang

**Affiliations:** 1 Department of Head and Neck Oncology, Affiliated Hospital of Zunyi Medical University, Zunyi, 563000 Guizhou Province People’s Republic of China; 2 Department of Head and Neck Oncology, Zunyi Medical University, Zunyi, 563000 Guizhou Province People’s Republic of China

**Keywords:** Glioma, Tumour-infiltrating immune cells (TIICs), CIBERSORT, Prognosis

## Abstract

**Object:**

Glioma is a common malignant tumours in the central nervous system (CNS), that exhibits high morbidity, a low cure rate, and a high recurrence rate. Currently, immune cells are increasingly known to play roles in the suppression of tumourigenesis, progression and tumour growth in many tumours. Therefore, given this increasing evidence, we explored the levels of some immune cell genes for predicting the prognosis of patients with glioma.

**Methods:**

We extracted glioma data from The Cancer Genome Atlas (TCGA). Using the Cell-type Identification by Estimating Relative Subsets of RNA Transcripts (CIBERSORT) algorithm, the relative proportions of 22 types of infiltrating immune cells were determined. In addition, the relationships between the scales of some immune cells and sex/age were also calculated by a series of analyses. A *P*-value was derived for the deconvolution of each sample, providing credibility for the data analysis (*P* < 0.05). All analyses were conducted using R version 3.5.2. Five-year overall survival (OS) also showed the effectiveness and prognostic value of each proportion of immune cells in glioma; a bar plot, correlation-based heatmap (corheatmap), and heatmap were used to represent the proportions of immune cells in each glioma sample.

**Results:**

In total, 703 transcriptomes from a clinical dataset of glioma patients were drawn from the TCGA database. The relative proportions of 22 types of infiltrating immune cells are presented in a bar plot and heatmap. In addition, we identified the levels of immune cells related to prognosis in patients with glioma. Activated dendritic cells (DCs), eosinophils, activated mast cells, monocytes and activated natural killer (NK) cells were positively related to prognosis in the patients with glioma; however, resting NK cells, CD8^+^ T cells, T follicular helper cells, gamma delta T cells and M0 macrophages were negatively related to prognosis in the patients with glioma. Specifically, the proportions of several immune cells were significantly related to patient age and sex. Furthermore, the level of M0 macrophages was significant in regard to interactions with other immune cells, including monocytes and gamma delta T cells, in glioma tissues through sample data analysis.

**Conclusion:**

We performed a novel gene expression-based study of the levels of immune cell subtypes and prognosis in glioma, which has potential clinical prognostic value for patients with glioma.

## Background

Accumulating studies have revealed that glioma is associated with high mortality, a high recurrence rate and a poor prognosis [1]. Although significant advances in the treatment of gliomas, including surgery, radiotherapy and chemotherapy, have occurred, the prognosis of glioma remains unsatisfactory, with the average survival of glioblastoma (GBM) patients being 15 months [2]. It still seems difficult for patients to comply with the treatment for glioma. Thus, there is an urgent need for researchers to develop novel strategies for glioma treatments.

Immune cells, as the base units of the immune system, in analysed samples are often heterogeneous with respect to cell subsets. In addition, extracted cell subset-specific information can be determined directly from heterogeneous samples via computational deconvolution techniques, such as the Cell-type Identification by Estimating Relative Subsets of RNA Transcripts (CIBERSORT) algorithm, thereby capturing both cell-centred and whole-system level contexts. Researchers have conducted numerous studies to verify the effectiveness of computational methods. The composition of immune cells in cancer tissues has been well validated and successfully evaluated by flow cytometry and other approaches [3]. Tumour-infiltrating immune cells (TIICs) include immune cells that migrate from the periphery to tumour tissues and exert a positive or negative effect; these cells have vital functional roles in promoting and/or regulating tumour progression and growth [4]. According to the varieties of cells, combined with their functional interactions, immune cells can play a main role in resisting tumour growth or in accelerating tumour growth in patients through their behaviours, such as defending or obliterating potential hazards [5]. In malignant gliomas, the immune system consists of several components, such as macrophages, natural killer (NK) cells, T cells, activated dendritic cells (DCs), eosinophils, activated mast cells, and monocytes. Various cytokines and chemokines are produced by these intratumoural immune cells, and these molecules are necessary for infiltrating immune cells to play inflammatory or anti-inflammatory roles with strong influences on glioma progression and resistance to therapeutic intervention [6]. Some studies have shown that microglia attract T-regulated lymphocytes to tumour sites, inhibit NK cell-mediated cytotoxicity, and block the functions of cytotoxic CD8^+^ T cells and the activation of tumour-reactive CD4^+^ T helper cells. With increases in tumour grade, the proportions of both CD8^+^ and CD4^+^ tumour-infiltrating T cells improve. In addition, patient survival may be improved by increasing the numbers of CD3^+^ and CD8^+^ cells but not CD4^+^ cells in tumours [7]. Compared to glioma patients with few CD8^+^ cells, patients with numerous CD8^+^ T cells at the time of diagnosis always have better survival [8]. Wu et al. recognized a significant difference between nontumour and GBM samples in several immune checkpoint modulators based on the expression levels of the corresponding genes. These differences could provide a valuable resource for identifying the involvement of these modulators in tumour escape mechanisms and the response to therapy in GBM [9].

Recently, significant advances have been made in immune cell infiltration into central nervous system (CNS) tumours, but the functions of these immune cells in tumour initiation and immune defence or tolerance still remain poorly understood. Some results have shown that blocking the programmed cell death-1 (PD-1)/programmed cell death-Ligand 1 (PD-L1) pathway in melanoma with brain metastasis may achieve a clinical cure through the roles of antibodies [10, 11]. This finding also suggests some novel therapeutics for tumours.

Over the past few years, several studies have addressed the abilities of immunotherapies, including (but not limited to) antibody-dependent cellular cytotoxicity (ADCC), chimeric antigen receptor T cell (CAR-T) therapy, cytokine treatment, cancer vaccination, checkpoint blockade, oncolytic virus treatment, and DC therapy. Immune cells, which are exposed to many cytokines and chemokines, are shown to be involved in the progression, invasion and therapeutic resistance of glioma through inflammatory responses or anti-inflammatory functions [6]. TIICs are likely to be effective targets for drugs to improve clinical outcomes.

In this study, we summarized current information about 22 kinds of TIICs generally recognized in the field that may prevent and/or boost the progression of glioma, as well as their proportions related to prognosis in glioma patients.

## Methods

### Workflow presentation

**Fig. 1:**
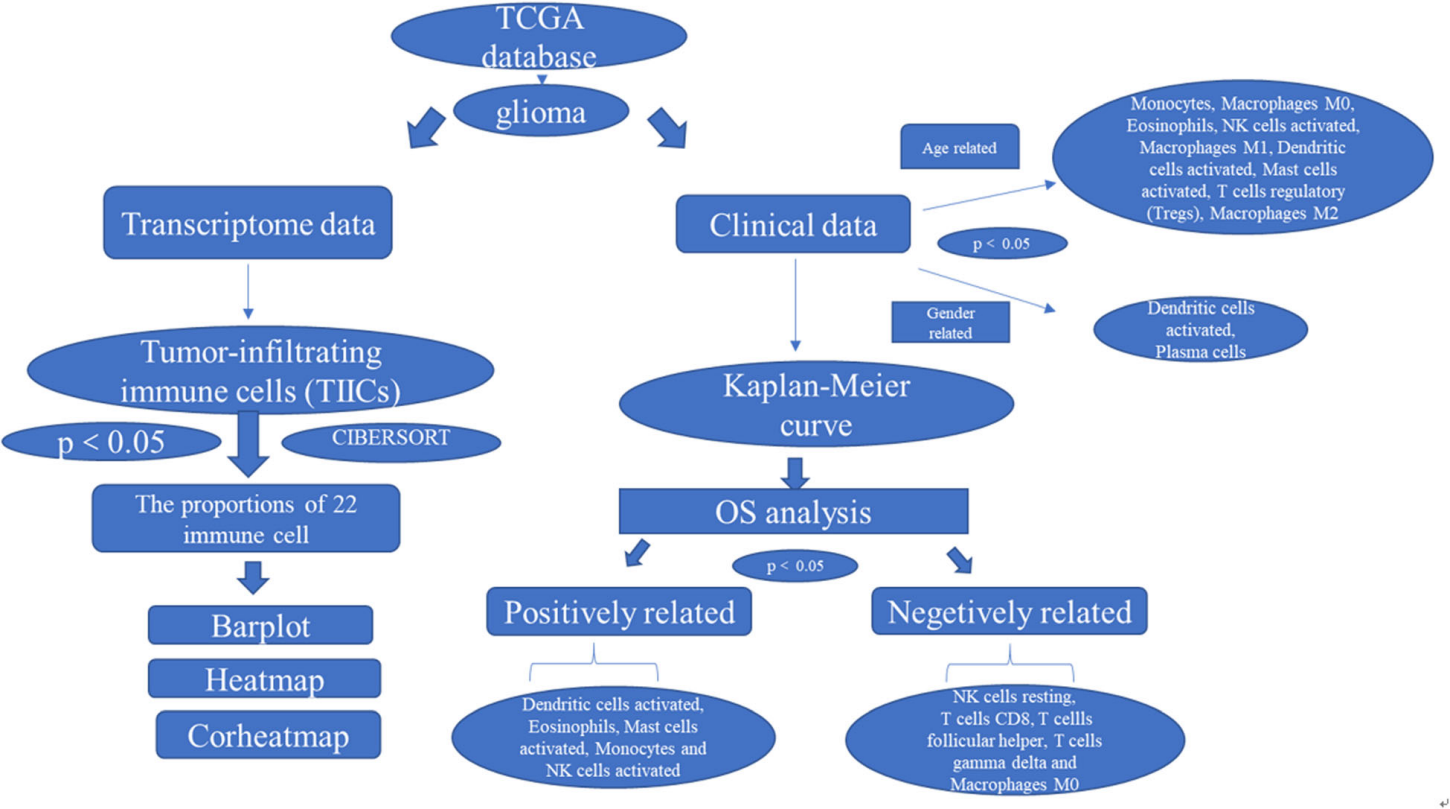
Genomic and transcriptomic data and clinical glioma information were extracted from the TCGA database. The proportions of immune cells in each glioma sample are displayed in a bar plot, corheatmap, and heatmap generated by using CIBERSORT. The associations between immune cell infiltrates and corresponding disease-free survival were evaluated by Kaplan-Meier analysis

We comparatively operated the CIBERSORT algorithm to analyse 703 cases from a TCGA dataset. Using the CIBERSORT algorithm, the relative proportions of 22 types of infiltrating immune cells were extracted. After combining the proportion data with clinical data, the relationships between the proportions of immune cells and the age or sex of the patients with glioma were analysed for statistically significant differences. A *P*-value was derived for the deconvolution of each sample, providing credibility for the results (*P* < 0.05). All analyses were conducted using R version 3.5. The effectiveness and prognostic value of each proportion of immune cells in glioma were confirmed by evaluating 5-year overall survival (OS); a bar plot, correlation-based heatmap (corheatmap), and heatmap were used to represent the proportions of immune cells in each sample of glioma (Fig. 1).

### Data acquisition

The expression profiles of immune cells and corresponding prognostic information of glioma patients were drawn from 703 samples (698 glioma samples vs. 5 normal control samples) in the TCGA. Among these patients, GBM and low-grade glioma (LGG) were included in the clinical pathology type. The expression profile of each sample and corresponding clinical dataset were logically organized. Second, there were strict exclusion criteria covering vague datasets for age, clinical pathology type, and time of disease progression. For the clinical data, there were a total of 1108 patients with G2/G3 disease (248 of them are G2), consisting of 459 women and 649 men with an age range of 10 to 89 years (590 of them were older than 50 years old). Among these patients, 559 died with their lifespan post-diagnosis ranging from 3 to 5166 days.

### CIBERSORT and assessment of TIICs

CIBERSORT, a computational method, is a deconvolution algorithm based on gene expression that was reported to predict the fractions of multiple cell types in the gene expression profiles (GEPs) of admixtures [12, 13]. The cellular composition of complex tissues can be estimated based on standardized gene expression data, which indicates the abundances of specific cell types [14–16]. For this study, the gene composition of each cell was determined by calculating the expression level of each gene in each immune cell, thereby performing a gene expression group analysis of 22 kinds of immune cells. In other words, CIBERSORT transformed the expression of genes into the levels of immune cells by analysing the compositions and proportions of 22 kinds of TIICs in tumour tissue samples.

A *P*-value was also derived for the deconvolution of each sample. Using the filtered data, the proportions of immune cells in each glioma sample were displayed in the form of a bar plot, corheatmap, and heatmap.

### Statistical analysis

In the survival analysis, CIBERSORT and a *P-*value < 0.05 were needed. Relationships between inferred percentages of immune cell varieties and survival are shown in a diagram. Kaplan-Meier curves showed the relationships between immune cell infiltrates and homologous disease-free survival. All analyses were conducted using R version 3.5.2, and *P* values < 0.05 were considered statistically significant.

## Results

### The distribution of immune infiltration in glioma

**Fig. 2:**
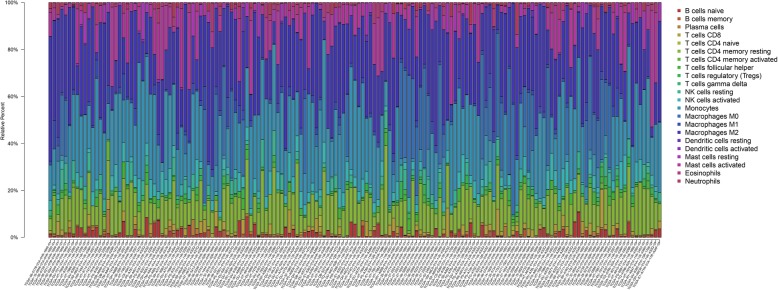
The proportions of immune cells in each glioma sample are indicated with different colours, and the lengths of the bars in the bar chart indicate the levels of the immune cell populations

**Fig. 3:**
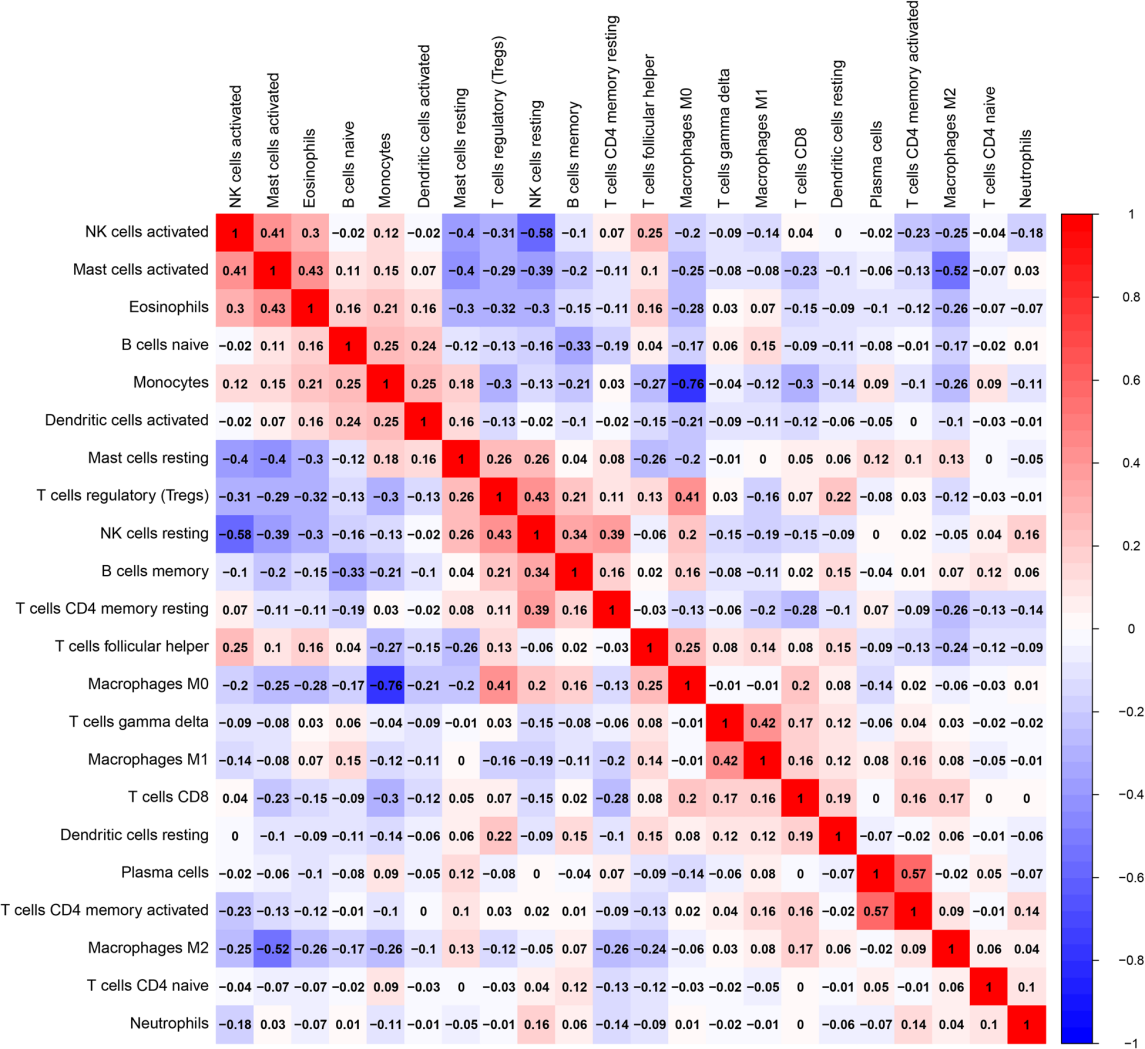
Correlation matrix for all 22 immune cell proportions. Some immune cells were negatively related, represented in blue, and others were positively related, represented in red. The darker the colour, the higher the correlation was (*P* < 0.05)

**Fig. 4:**
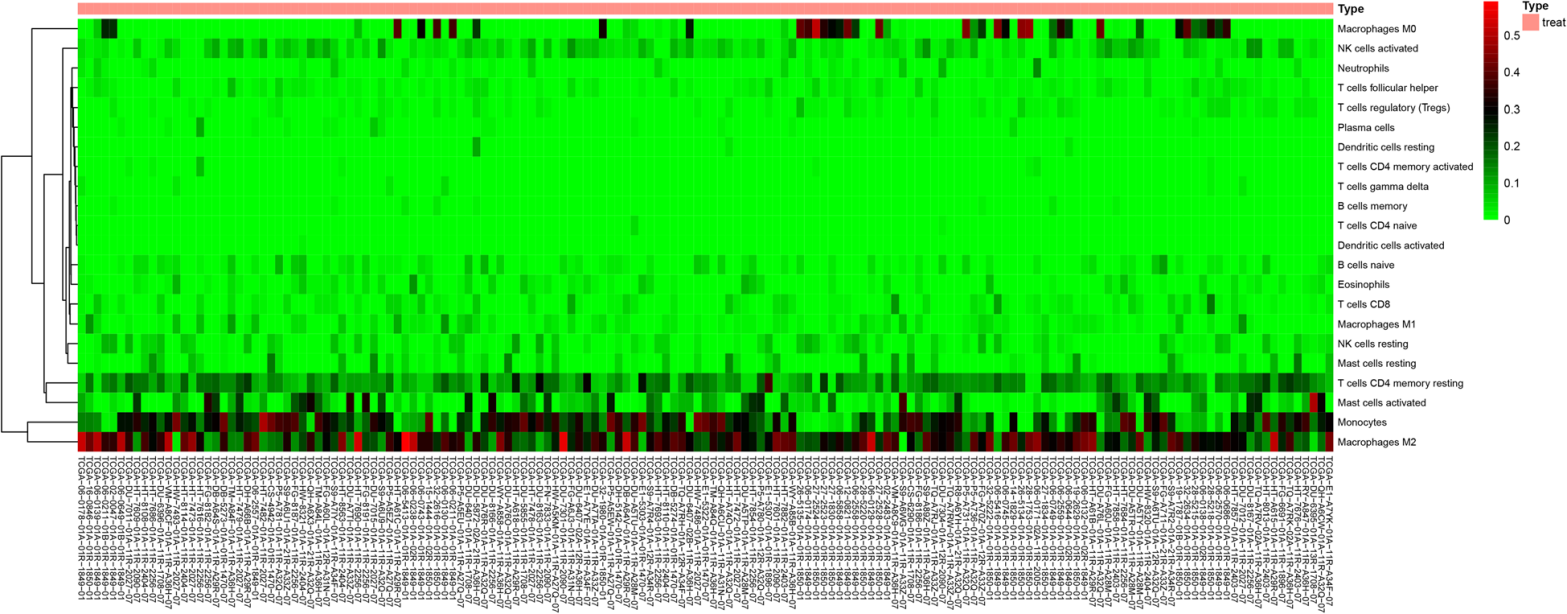
Heat map of the 22 immune cell proportions. Each column represents a sample, and each row represents one of the immune cell populations. The levels of the immune cell populations are shown in different colours, which transition from green to red with increasing proportions

The distribution of immune infiltration in glioma has not been fully displayed owing to technical limitations and small cell populations. We first explored immune infiltration in glioma tissue in 22 subpopulations of immune cells by using the CIBERSORT algorithm. Figure 2 shows the proportions of immune cells in each glioma sample in different colours, and the lengths of the bars in the bar chart indicate the levels of the immune cell populations. Next, we inferred that divergence in TIIC proportions might serve as an essential characteristic of individual differences and have prognostic value. From the chart, we identified that glioma tissue had relatively high percentages of M0, M1 and M2 macrophages and monocytes, accounting for approximately 60% of the 22 subpopulations of immune cells. Conversely, B cell and neutrophil percentages were relatively low, accounting for approximately 10% (Fig. 2). Indeed, the percentages of different TIICs subsets were not obviously correlated, as shown by the corheatmap (Fig. 3). The populations with a significantly negative relation included activated mast cells and M2 macrophages (− 0.52); monocytes and M0 macrophages (− 0.76); and activated NK cells and resting mast or NK cells (− 0.58). The populations with a significantly positive relation were eosinophils and activated mast cells (0.43); activated NK cells and activated mast cells (0.41) or eosinophils (0.3); gamma delta T cells and M0 macrophages (0.42); and resting NK cells and regulatory T cells (Tregs) (0.43). In Fig. 4, using unsupervised hierarchical clustering according to the above cell subsets, the levels of M2 macrophages, monocytes, activated mast cells and resting CD4^+^ memory T cells were relatively high in the samples of tumours included in the heatmap. Together, as a regulated process, abnormal immune cell infiltration in glioma and its heterogeneity may have special guiding significance in the clinic.

### The clinical features of the dataset and immune cells in glioma

**Fig. 5:**
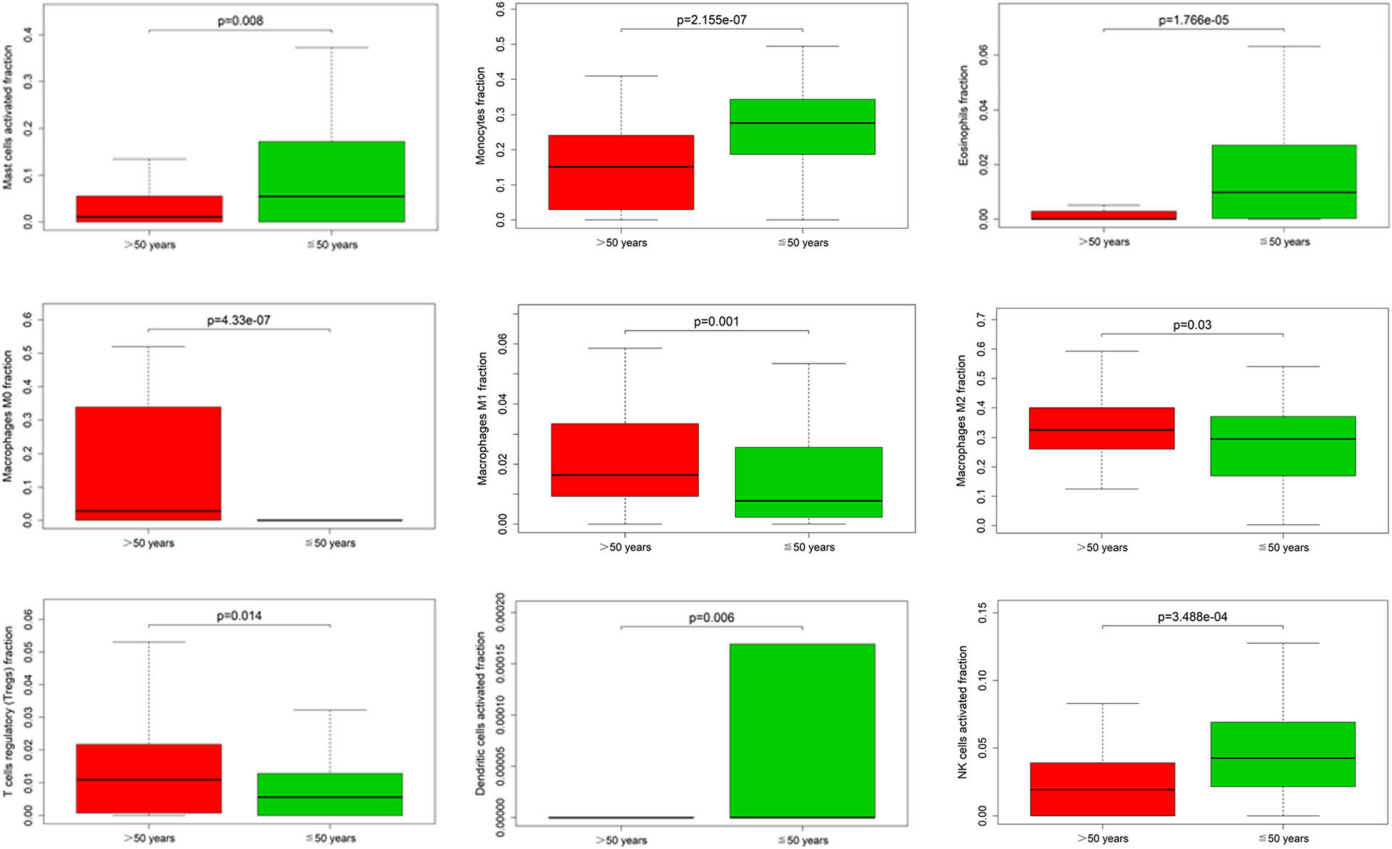
These genes were obviously related to age in patients with glioma (50 years old as the age cut-off) (*P* < 0.05)

**Fig. 6:**
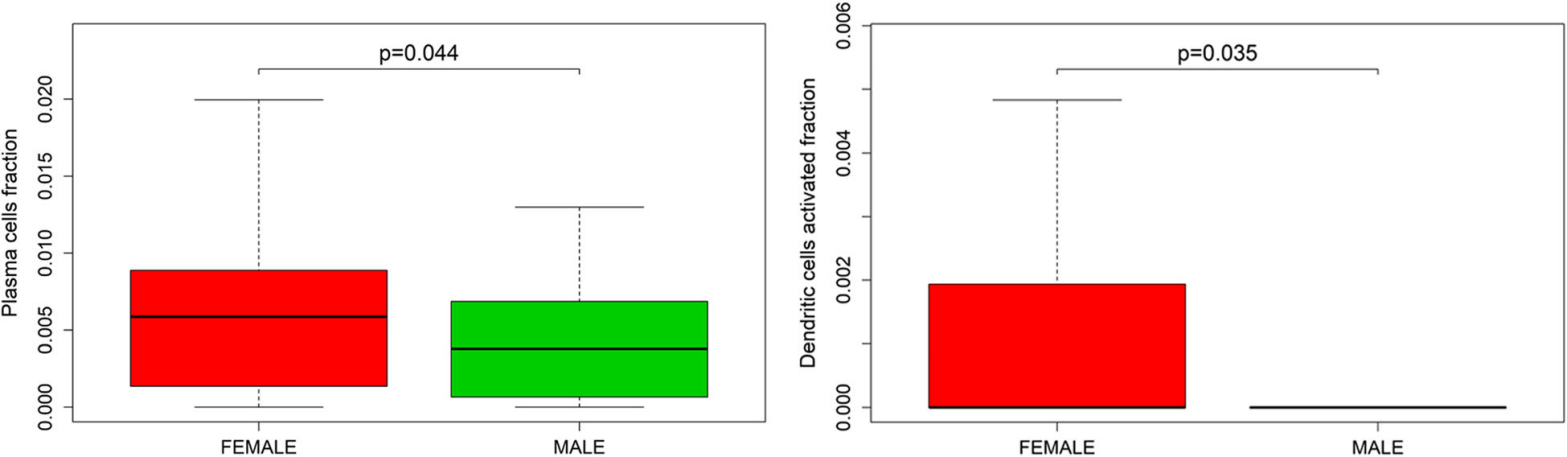
These genes were obviously related to sex in patients with glioma (*P* < 0.05)

In this study, we have drawn clinical datasets of glioma with some clinical features (age, sex, clinical pathology type, and the time of disease progression) from the TCGA database. After performing analytical studies, we found that the proportions of several immune cells were significantly related to patient age and sex but not to clinical pathology type. Monocytes, M0 macrophages, eosinophils, activated NK cells, M1 macrophages, activated DCs, activated mast cells, Tregs, and M2 macrophages were observably related to patient age in glioma (50 years old as the age cut-off). Among these populations, monocytes, eosinophils, activated NK cells, and activated mast cells were found in high proportions in the patients with glioma less than or equal to 50 years old. The other populations were found at high levels in the patients over 50 years old (Fig. 5). In addition, activated DCs and plasma cells usually were found at high levels in female patients with glioma (*P* < 0.05) (Fig. 6).

### The relationships between prognosis and TIICs in glioma

**Fig. 7:**
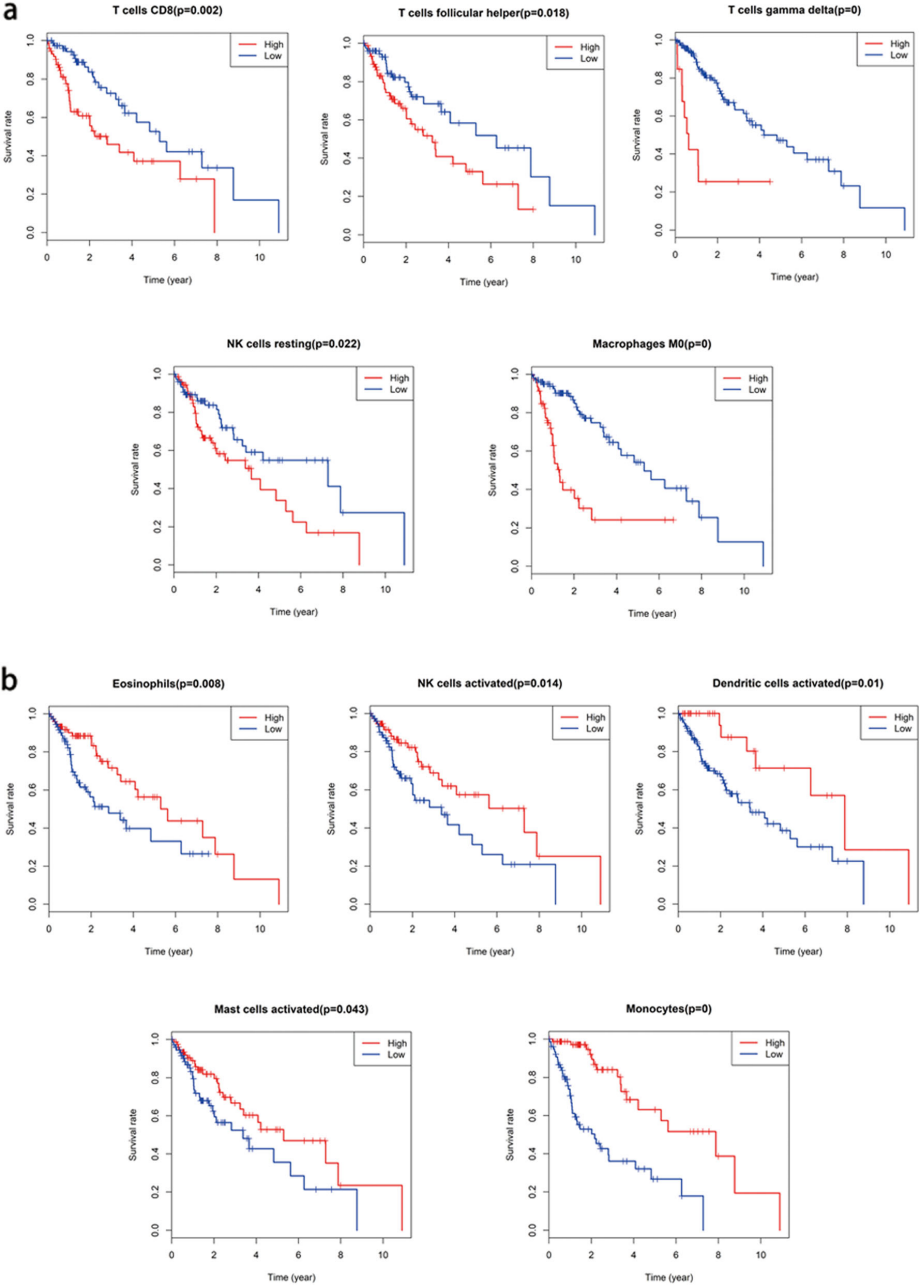
Survival curves for the specific immune cell populations whose levels showed significant correlations with survival are shown (*P* < 0.05). Red lines indicate high expression, and blue lines indicate low expression. **a**. These five immune cell populations were positively related to 5-year OS. **b**. These five immune cell populations were negatively related to 5-year OS

From our study, prognosis was partly reflected by discrepancies in TIIC subpopulation levels among individuals. Kaplan-Meier curve analysis for the above-identified TIIC subsets and others are shown in Fig. 7. Activated DCs, eosinophils, activated mast cells, monocytes and activated NK cells were positively related to 5-year OS in patients with glioma (Fig. 7a). However, resting NK cells, CD8^+^ T cells, T follicular helper cells, gamma delta T cells and M0 macrophages were negatively related to 5-year OS (Fig. 7b). These findings mean that TIIC subpopulations could provide additional prognostic value for the operating therapeutic method.

## Discussion

Glioma is one of the most aggressive brain tumours. Due to infiltration of adjacent brain tissues, gliomas tend to be incurable, even when treatments are combined. Emerging evidence suggests that TIICs play main roles in the diagnosis and treatment of patients with glioma.

As an advancement in molecular research, TIICs can promote and/or regulate tumour progression and growth by means of the types of cells and their interactions. Recently, in CNS tumours, many developments have been achieved for immune cell infiltrates, but their roles in tumour origination and patient prognosis remain poorly understood. Therefore, we focused on a gene expression-based study of immune infiltration and the clinical prognosis of glioma to offer a possible immune treatment.

Monocytes are found in the bone marrow, blood, and spleen of vertebrates at the time of homeostasis and can be recruited to injured or infected tissue to function as effectors and particularly as progenitors of DCs and macrophages [17, 18]. Monocytes exist in three forms, persisting as monocytes, repolarizing into a different monocyte subset, and differentiating into macrophages [19].

During tissue injury and regeneration, monocytes and macrophages can be the first reactors among immune cells [20]. They are regulators of inflammation and the immune response, representing the critical parts of the immune system. In addition, during infection or inflammation, monocytes mobilize from the bone marrow, transit to the required destination and differentiate into effector cells, and monocytes may perform multiple roles depending on the local tissue environment, which makes them an important component of the body’s immune defence system. Moreover, in tissue homeostasis, development, and tissue repair following injury, macrophages also have various roles. During an infection or inflammatory reactions, adult bone marrow monocytes can undergo self-replication and give rise to tissue-resident macrophages [21]. Wang et al. found a decrease in invading monocyte numbers and a subtype-dependent increase in the numbers of macrophages/microglia upon glioma recurrence according to a gene signature-based tumour microenvironment inference. Hypermutation at diagnosis or recurrence of glioma was associated with CD8^+^ T cell enrichment. Notably, M2 macrophages were also associated with short-term relapse after radiation therapy in glioma [22]. Glioma-associated macrophages/monocytes (GAMPs), as tumour-supporting cells, can invade into gliomas from the blood circulation, which has been shown to promote glioma growth and invasion [23]. Given the significantly negative relationship between monocytes and M0 macrophages, which has a ratio of − 0.76, in addition to macrophages M0 being negatively related to OS, we hypothesized that M0 macrophages play an important role in the development of glioma following the transformation of monocytes.

Gamma delta T cells, which are a small population within the overall T lymphocyte population (0.5–5%), have a variable tissue distribution in the body [24]. They act as a line of primary defence to resist invading pathogens during early life, secreting various chemokines to attract neutrophils to the site of inflammation and assisting in pathogen clearance [25]. Bryant et al. showed that expanded/activated gamma delta T cells from both patients and healthy volunteers killed the GBM cell lines D54, U373, and U251, as well as primary GBM cells, without cytotoxicity to primary astrocyte cultures. In addition, gamma delta T cell depletion and impaired function occurred prior to or concurrent with tumour growth in GBM patients [26]. In our data analysis, gamma delta T cells were negatively related to OS, while showing a positive correlation with M0 macrophages at a ratio of 0.42. This finding could reveal that gamma delta T cells and M0 macrophages promote development via a synergistic effect.

NK cells exert cytolytic activity by secreting tumour necrosis factor (TNF) and interferon (IFN) to kill susceptible target cells. They integrate or engage many signalling pathways to distinguish between normal and abnormal cells (infected or transformed), which can protect healthy cells from NK cell-mediated lysis by signalling via NK cell inhibitory receptors activated by major histocompatibility complex (MHC) class I ligands [27–29]. Previous research has reported that resting NK cells, which secrete tumour necrosis factor α (TNF-α) and interferon γ (IFN-γ), can kill target cells by specific paired receptor-ligand binding [30]. As GBM tumours are frequently infiltrated by NK cells, these immune cells are actively suppressed by GBM cells through the expression of ligands for inhibitory NK cell receptors and factors such as TGF-β [31]. GBM cells also inhibit NK cell activity indirectly through myeloid cells that induce downregulation of the activating NK cell receptor NKG2D [32]. Therefore, according to our data analysis, resting NK cells, in contrast to activated NK cells, were negatively related to OS and might play a role in progressive glioma. From those findings, we may infer that NK cells indeed have the ability to eliminate tumour tissue through immune function.

DCs, which participate in the regulation of T cell immunity, are potent antigen-presenting cells. They enhance the immunogenicity of special antigens in patients and are increasingly used in vaccination procedures [33]. DCs can induce tumour-specific cytotoxic T lymphocytes and enhance NK cell immunity [34]. Baur and colleagues showed that the function of DCs could be negatively affected by denileukin diftitox, which prevented the induction of tumour-specific cytotoxic T lymphocytes by inducing a tolerogenic phenotype in DCs and by promoting the survival of non-activated Tregs [35]. This finding reminds us that DCs may play a significant role in glioma by activating T lymphocytes.

Eosinophils contain a number of cytotoxic compounds in their granules and are associated with an improved prognosis in tumour patients by affecting tumour cell viability [36]. Previous studies have shown that eosinophils accumulate in various human CNS disorders, including tumours of the brain (neuroblastoma, leiomyoma, and GBM) [37]. In addition, eosinophilic meningitis was identified in a case of disseminated GBM [38]. In an in vivo murine model, eosinophils were shown to be recruited to necrotic tissue [39], which is also a primary determinant of human GBM [40]. In some clinical trials, enhanced GBM patient survival was associated with tissue eosinophilia found after postoperative treatments with interleukin (IL)-2) [37]. Youngil et al. also found that DCs might contribute to ongoing eosinophilic inflammation in asthmatic airways and vice versa [41]. In our research, activated DCs and eosinophils were positively related to the 5-year OS of patients with glioma, and they were related to each other with a ratio of 0.16. All of these findings make us consider that DCs and eosinophils are cooperative partners in the killing of glioma cells.

Immune checkpoints provide a common mechanism for different cancers to avoid immunosurveillance and have roles in the immune system. In lung cancer, anti-CTLA-4 and anti-PD-1/PD-L1 blocking antibodies have shown therapeutic success. In addition, there are also identifying markers of early response in lung cancer, such as the TCR repertoire, the CD4^+^/CD8^+^ T cell profile, the cytokine signature, and immune checkpoint molecule expression in tumour cells, macrophages or T cells [42]. In breast cancer, immune suppressor cells, for example, myeloid-derived suppressor cells (MDSCs) and M2 macrophages, can release suppressive factors, such as IL-10, indoleamine dioxygenase 1 (IDO1), reactive oxygen species (ROS) and nitric oxide (NO), to suppress T and NK cell functions and promote tumour growth and metastasis [43, 44]. Another factor, PD-L1, is expressed in most breast cancers, and high levels of PD-L1 expression are associated with poor OS in breast cancer [45].

In conclusion, different types of infiltrating immune cells vary not only among different types of cancers but also in the same type of tumour or at different time points in the same patient. Thus, it is imperative to explore the heterogeneity of immune cell indicators for prognostic prediction in glioma and even for individualized treatment in the future.

## Conclusion

In this study, we analysed the latest data for 22 kinds of TIICs generally recognized in the field and the effects of their levels on the prognosis of glioma patients, which may offer help in the development of glioma treatments.

## Data Availability

The datasets used and/or analysed during the current study are available from the corresponding author on reasonable request.

## Abbreviations

ADCC: Antibody dependent cellular cytotoxicity
CAR-T: Chimeric antigen receptor T-cell therapy
CNS: Central nervous system
NK: Natural Killer
OS: Overall survival
TCGA: The Cancer Genome Atlas
TIIC: Tumor-infiltrating immune cells

## References

[1] Bie L, Zhao G, Cheng P, Rondeau G, Porwollik S, Ju Y (2011). The accuracy of survival time prediction for patients with glioma is improved by measuring mitotic spindle checkpoint gene expression. PLoS One.

[2] Omar AI (2014). Tumor treating field therapy in combination with bevacizumab for the treatment of recurrent glioblastoma. J Vis Exp.

[3] Cackowski FC, Wang Y, Decker JT, Sifuentes C, Weindorf S, Jung Y (2019). Detection and isolation of disseminated tumor cells in bone marrow of patients with clinically localized prostate cancer. Prostate..

[4] Whiteside TL (2008). The tumor microenvironment and its role in promoting tumor growth. Oncogene.

[5] Taphoorn MJ, Klein M (2004). Cognitive deficits in adult patients with brain tumours. Lancet Neurol.

[6] Boussiotis VA, Charest A (2018). Immunotherapies for malignant glioma. Oncogene..

[7] Heimberger AB, Abou-Ghazal M, Reina-Ortiz C, Yang DS, Sun W, Qiao W (2008). Incidence and prognostic impact of FoxP3+ regulatory T cells in human gliomas. Clin Cancer Res.

[8] Alexiou GA, Vartholomatos G, Karamoutsios A, Batistatou A, Kyritsis AP, Voulgaris S (2013). Circulating progenitor cells: a comparison of patients with glioblastoma or meningioma. Acta Neurol Belg.

[9] Wu S, Yang W, Zhang H, Ren Y, Fang Z, Yuan C (2019). The prognostic landscape of tumor-infiltrating immune cells and immune checkpoints in glioblastoma. Technol Cancer Res Treat.

[10] Wolchok JD, Chiarion-Sileni V, Gonzalez R, Rutkowski P, Grob JJ, Cowey CL (2017). Overall survival with combined nivolumab and ipilimumab in advanced melanoma. N Engl J Med.

[11] Long GV, Atkinson V, Lo S, Sandhu S, Guminski AD, Brown MP (2018). Combination nivolumab and ipilimumab or nivolumab alone in melanoma brain metastases: a multicentre randomised phase 2 study. Lancet Oncol.

[12] Shen-Orr Shai S, Gaujoux Renaud (2013). Computational deconvolution: extracting cell type-specific information from heterogeneous samples. Current Opinion in Immunology.

[13] Newman AM, Liu CL, Green MR, Gentles AJ, Feng W, Xu Y (2015). Robust enumeration of cell subsets from tissue expression profiles. Nat Meth.

[14] Ge P, Wang W, Lin L, Zhang G, Gao Z, Tang Z (2019). Profiles of immune cell infiltration and immune-related genes in the tumor microenvironment of colorectal cancer. Biomed Pharmacother.

[15] Zhang S, Zhang E, Long J, Hu Z, Peng J, Liu L (2019). Immune infiltration in renal cell carcinoma. Cancer Sci.

[16] Yang X, Shi Y, Li M, Lu T, Xi J, Lin Z (2019). Identification and validation of an immune cell infiltrating score predicting survival in patients with lung adenocarcinoma. J Transl Med.

[17] Shi C, Pamer EG (2011). Monocyte recruitment during infection and inflammation. Nat Rev Immunol.

[18] Yona S, Kim KW, Wolf Y, Mildner A, Varol D, Breker M (2013). Fate mapping reveals origins and dynamics of monocytes and tissue macrophages under homeostasis. Immunity.

[19] Dal-Secco D, Wang J, Zeng Z, Kolaczkowska E, Wong CH, Petri B (2015). A dynamic spectrum of monocytes arising from the in situ reprogramming of CCR2 þ monocytes at a site of sterile injury. J Exp Med.

[20] Bergmann CE, Hoefer IE, Meder B, Roth H, van Royen N, Breit SM (2006). Arteriogenesis depends on circulating monocytes and macrophage accumulation and is severely depressed in op/op mice. J Leukoc Biol.

[21] Ray R, Rai V (2017). Lysophosphatidic acid converts monocytes into macrophages in both mice and humans. Blood.

[22] Wang Q, Hu B, Hu X, Kim H, Squatrito M, Scarpace L (2017). Tumor evolution of glioma intrinsic gene expression subtype associates with immunological changes in the microenvironment. Cancer Cell.

[23] Vinnakota K, Hu F, Ku MC, Georgieva PB, Szulzewsky F, Pohlmann A (2013). Toll-like receptor 2 mediates microglia/brain macrophage MT1-MMP expression and glioma expansion. Neuro-Oncology.

[24] Carding SR, Kyes S, Jenkinson EJ, Kingston R, Bottomly K, Owen JJ (1990). Developmentally regulated fetal thymic and extrathymic T-cell receptor gamma delta gene expression. Genes Dev.

[25] Nakasone C, Yamamoto N, Nakamatsu M, Kinjo T, Miyagi K, Uezu K (2007). Accumulation of gamma/delta T cells in the lungs and their roles in neutrophil-mediated host defense against pneumococcal infection. Microbes Infect.

[26] Bryant NL, Suarez-Cuervo C, Gillespie GY, Markert JM, Nabors LB, Meleth S (2009). Characterization and immunotherapeuticn potential of gammadelta T-cells in patients with glioblastoma. Neuro-Oncology.

[27] Lanier LL (2005). NK cell recognition. Annu Rev Immunol.

[28] Chiesa S, Tomasello E, Vivier E, Vely F (2005). Coordination of activating and inhibitory signals in natural killer cells. Mol Immunol.

[29] Moretta L, Moretta A (2004). Unravelling natural killer cell function: triggering and inhibitory human NK receptors. EMBO J.

[30] Bryceson YT, March ME, Ljunggren HG, Long EO (2006). Synergy among receptors on resting NK cells for the activation of natural cytotoxicity and cytokine secretion. Blood..

[31] Crane CA, Han SJ, Barry JJ, Ahn BJ, Lanier LL, Parsa AT (2010). TGF-beta downregulates the activating receptor NKG2D on NK cells and CD8+ T cells in glioma patients. Neuro-Oncology.

[32] Crane CA, Austgen K, Haberthur K, Hofmann C, Moyes KW, Avanesyan L (2014). Immune evasion mediated by tumor-derived lactate dehydrogenase induction of NKG2D ligands on myeloid cells in glioblastoma patients. Proc Natl Acad Sci U S A.

[33] Banchereau J, Palucka AK (2005). Dendritic cells as therapeutic vaccines against cancer. Nat Rev Immunol.

[34] Lion E, Smits EL, Berneman ZN, Van Tendeloo VF (2012). NK cells: key to success of DC-based cancer vaccines?. Oncologist.

[35] Baur AS, Lutz MB, Schierer S (2013). Denileukin diftitox (ONTAK) induces a tolerogenic phenotype in dendritic cells and stimulates survival of resting Treg. Blood.

[36] Lotfi R, Lee JJ, Lotze MT (2007). Eosinophilic granulocytes and damage associated molecular pattern molecules (DAMPs): role in the inflammatory response within tumors. J Immunother.

[37] Hayes RL, Arbit E, Odaimi M, Pannullo S, Scheff R, Kravchinskiy D (2001). Adoptive cellular immunotherapy for the treatment of malignant gliomas. Crit Rev Oncol Hematol.

[38] Defendini R, Hunter SB, Schlesinger EB, Leifer E, Rowland LP (1981). Eosinophilic meningitis in a case of disseminated glioblastoma. Arch Neurol.

[39] Cormier SA, Taranova AG, Bedient C, Nguyen T, Protheroe C, Pero R (2006). Pivotal advance: eosinophil infiltration of solid tumors is an early and persistent inflammatory host response. J Leukoc Biol.

[40] Tehrani M, Friedman TM, Olson JJ, Brat DJ (2008). Intravascular thrombosis in central nervous system malignancies. A potential role in astrocytoma progression to glioblastoma. Brain Pathol.

[41] Koh YI, Lee J-B, Lee S-R, Ji S-G, Choi IS (2005). Relationship between dendritic cells and activated eosinophils in induced sputum of asthmatics. J Korean Med Sci.

[42] Janakiram M, Pareek V, Cheng H, Narasimhulu DM, Zang X (2016). Immune checkpoint blockade in human cancer therapy: lung cancer and hematologic malignancies. Immunotherapy..

[43] Ward R, Sims AH, Lee LC, Wynne L, Yusuf H, Gregson H (2015). Monocytes and macrophages, implications for breast cancer migration and stem cell-like activity and treatment. Oncotarget.

[44] Lindau D, Gielen P, Kroesen M, Wesseling P, Adema GJ (2013). The immunosuppressive tumour network: myeloid-derived suppressor cells, regulatory T cells and natural killer T cells. Immunology.

[45] Muenst S, Schaerli AR, Gao F, Däster S, Trella E, Droeser RA (2014). Expression of programmed death ligand 1 (PD-L1) is associated with poor prognosis in human breast cancer. Breast Cancer Res Treat.

